# Neuromodulatory Effects of Auditory Training and Hearing Aid Use on Audiovisual Speech Perception in Elderly Individuals

**DOI:** 10.3389/fnagi.2017.00030

**Published:** 2017-02-21

**Authors:** Luodi Yu, Aparna Rao, Yang Zhang, Philip C. Burton, Dania Rishiq, Harvey Abrams

**Affiliations:** ^1^Department of Speech-Language-Hearing Sciences and Center for Neurobehavioral Development, University of MinnesotaMinneapolis, MN, USA; ^2^Department of Speech and Hearing Sciences, Arizona State UniversityTempe, AZ, USA; ^3^Office of the Associate Dean for Research, College of Liberal Arts, University of MinnesotaMinneapolis, MN, USA; ^4^Department of Speech Pathology and Audiology, University of South AlabamaMobile, AL, USA

**Keywords:** brain plasticity, auditory training, hearing aid, audiovisual integration, speech perception, fMRI, functional connectivity

## Abstract

Although audiovisual (AV) training has been shown to improve overall speech perception in hearing-impaired listeners, there has been a lack of direct brain imaging data to help elucidate the neural networks and neural plasticity associated with hearing aid (HA) use and auditory training targeting speechreading. For this purpose, the current clinical case study reports functional magnetic resonance imaging (fMRI) data from two hearing-impaired patients who were first-time HA users. During the study period, both patients used HAs for 8 weeks; only one received a training program named ReadMyQuips^TM^ (RMQ) targeting speechreading during the second half of the study period for 4 weeks. Identical fMRI tests were administered at pre-fitting and at the end of the 8 weeks. Regions of interest (ROI) including auditory cortex and visual cortex for uni-sensory processing, and superior temporal sulcus (STS) for AV integration, were identified for each person through independent functional localizer task. The results showed experience-dependent changes involving ROIs of auditory cortex, STS and functional connectivity between uni-sensory ROIs and STS from pretest to posttest in both cases. These data provide initial evidence for the malleable experience-driven cortical functionality for AV speech perception in elderly hearing-impaired people and call for further studies with a much larger subject sample and systematic control to fill in the knowledge gap to understand brain plasticity associated with auditory rehabilitation in the aging population.

## Introduction

Hearing loss is common among older people. Over 30% of the adult population between the ages of 65 and 74 and nearly 50% of people older than 75 have a hearing loss that affects communication and consequently psychosocial health (National Institute on Deafness and Other Communication Disorders, https://www.nidcd.nih.gov). Despite gains achieved through advanced signal processing technology of hearing aids (HAs), users report persistent problems in speech perception in the presence of noise relative to premorbid experience (Kochkin, 2007), and rehabilitative training has been proposed to address these problems (Boothroyd, 2007; Moore and Amitay, 2007).

A topic of current interest in audiology and aging neuroscience is the benefits and neuromodulatory effects from HA use and auditory training (Pichora-Fuller and Levitt, 2012; Anderson et al., 2013; Ferguson and Henshaw, 2015; Morais et al., 2015; Rao et al., 2017). Electroencephalography (EEG) studies have shown mixed results at the subcortical (Philibert et al., 2005; Dawes et al., 2013) and cortical levels (Bertoli et al., 2011; Dawes et al., 2014). Although functional magnetic resonance imaging (fMRI) can provide millimeter spatial resolution for investigating neuroanatomical basis of auditory plasticity (Hall, 2006), only one fMRI study has documented neuromodulatory effects after 3 months of HAs use in eight adults aged 30–53 who had congenital sensorineural hearing loss (SNHL; Hwang et al., 2006).

As speech perception is inherently a multi-sensory process (McGurk and MacDonald, 1976; see review in Rosenblum, 2008), aural rehabilitation involving speechreading can be designed to better utilize visual articulation cues. Speech training including visual articulation has been found to facilitate second language learning in adulthood (Zhang et al., 2009). In particular, addition of visual cues can improve speech recognition by 60% depending on the materials used (Erber, 1969; Summerfield, 1979; Middelweerd and Plomp, 1987; Bernstein et al., 2013), which is equivalent to an increase of 5–18 dB in signal-to-noise ratio (S/N). However, there has been no imaging data from individuals with age-related SNHL to elucidate the cortical mechanisms mediating the auditory rehabilitation process.

In this report, we present fMRI data from two patients with age-related SNHL to examine effects of HA use and audiovisual (AV) training. Our experiment adopted the well-known McGurk effect of perceiving a fused /da/ from visual articulation of /ga/ dubbed with /ba/ sound (McGurk and MacDonald, 1976). Previous research on normal hearing listeners has shown that posterior superior temporal gyrus (pSTS) as the cortical locus for McGurk perception (Beauchamp et al., 2010; Matchin et al., 2014), and activity within the left pSTS correlated with magnitude of the McGurk effect (Nath and Beauchamp, 2011, 2012). Moreover, connectivity between superior temporal sulcus (STS) and sensory regions were found to be dynamically correlated with S/N of the sensory input. Based on these findings and the exploratory nature of the current case report, we expect to see neuromodulatory effects associated with three regions of interest (ROIs) within the left hemisphere, including auditory ROI within Heschl’s gyrus, visual ROI within occipitotemporal lobe representing uni-sensory regions, and the AV ROI within pSTS.

## Patients and Methods

### Subjects and Hearing Aids

**Figure 1:**
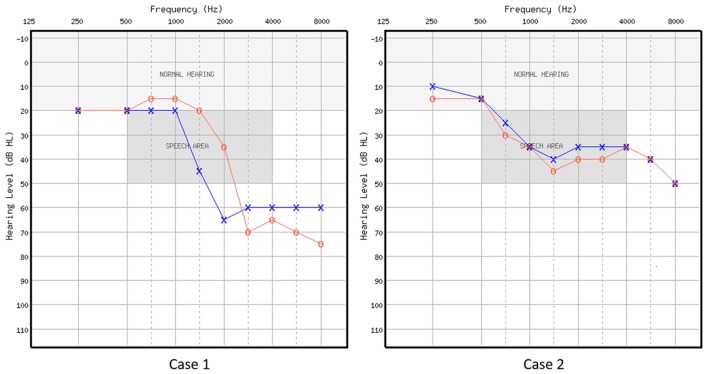
**Air-conduction audiometric thresholds in dB HL for the two cases.** Red circle represents right ear, and blue cross represents left ear.

Two volunteers were recruited from an audiology clinic. Both were part of a larger-scale behavioral study (Rishiq et al., 2016). Case 1 (C1) was a 68-year-old male with bilateral normal thresholds through 1 kHz, precipitously sloping to moderately-severe SNHL in the left ear and severe in the right ear. Case 2 (C2) was a 52-year-old female with bilateral mild to moderate relatively flat SNHL (for audiometric thresholds, see Figure 1 and Table 1). C1 only received HA trial, and C2 received HA trial as well as AV training. These treatment(s) were implemented in Rishiq et al. (2016) on HA use with and without ReadMyQuips^TM^ (RMQ) training, of which C1 and C2 were participants. Both patients were first-time HA users, native speakers of American English, right-handed as measured using the Edinburgh Handedness Inventory (EHI; Oldfield, 1971). Behavioral screening with a protocol from Nath and Beauchamp (2012) showed that neither of them was a perfect McGurk perceiver. Medical histories showed no cognitive, speech-language, or other chronic medical disorders. They passed the safety screening requirements for the fMRI procedure at the Center for Magnetic Resonance Research of the University of Minnesota, MN, USA and informed consent was obtained from each participant following a protocol approved by the Institutional Review Board of the University of Minnesota.

**Table 1:** **Air-conduction audiometric thresholds in dB HL for the two cases**.

Participant		Mean thresholds in dB HL									
Frequency		250	500	750	1000	1500	2000	3000	4000	6000	8000
Case 1	R	20	20	15	15	20	35	70	65	70	75
	L	20	20	20	20	45	65	60	60	60	60
Case 2	R	15	15	30	35	45	40	40	35	40	50
	L	10	15	25	35	40	35	35	35	40	50

*R stands for right ear, and L stands for left ear*.

Both patients were fitted with binaural three Series i110 receiver-in-the canal (RIC) 13 Starkey HAs (Eden Prairie, MN, USA) according to National Acoustic Laboratories Non-Linear two prescription targets, which were verified with real-ear probe microphone measurements. The participants wore the HAs for 1 week after which parameters were adjusted as needed based upon the participants’ feedback. Both patients wore the HAs for at least 6 h/day throughout the study period, which was verified using the HA data logging feature. C2 was instructed to use the computerized training program for at least 30 min/day for 5 days/week during the second 4 weeks of the whole 8-week study period. Compliance was logged daily using a journal.

### Training Program

The auditory rehabilitation used “RMQ”^1^. RMQ is a computerized program designed to improve speech understanding through AV training in the presence of background noise. RMQ training has been shown to improve HA users’ speech-in-noise perception as well as confidence in target detection in auditory selective attention task (Abrams et al., 2015; Rao et al., 2017).

### Stimuli and fMRI Data Collection

The event-related fMRI experiment contained the following stimuli presented in five runs: 50 auditory-only /ba/ and /ga/ syllables (AO condition), 50 visual-only /ba/ and /ga/ syllables (VO condition), 50 AV /ba/ and /ga/ syllables (congruent condition), 50 McGurk incongruent AV syllables (i.e., visual /ga/ with auditory /ba/; McGurk incongruent condition), 50 non-McGurk incongruent AV syllables (i.e., visual /ba/ with auditory /ga/; non-McGurk incongruent condition). Other than these, 25 AV /la/ syllables were presented randomly as decoy trials to maintain participant’s attention. The participant was instructed to watch and listen to the stimuli carefully and press a button whenever hearing a /la/ sound. Each 1-s trial contained one syllable with random inter-stimulus interval of 2 s, 4 s and 6 s. Auditory stimuli were delivered through Avotec Silent Scan^®^ headphones (Avotec, Inc., Stuart, FL, USA) at the participants’ comfortable level (about 108 dB SPL). Visual stimuli were presented through a projector screen.

C1’s fMRI data were collected before (pretest) and after 8 weeks (posttest) of HA use. The same time frame of data collection applied to C2 with the identical protocol. fMRI scans were acquired using Siemens 3-Tesla MR Scanner with a 12-channel head coil. For each session, the participants underwent eight scans: a T1-weighted MPRAGE anatomical scan to obtain structural volume (TR = 2600 ms, TE = 3.02 ms, flip angle = 8°) with 176 sagittal slices; an independent functional localizer for identification of ROIs; five main experimental T2*-weighted gradient-echo-planar imaging (EPI) scans for detection of McGurk related BOLD effects; a reversed-phase EPI scan for distortion correction (Smith et al., 2004). EPI parameters were as follows: TR = 2000 ms, TE = 28 ms, flip angle = 80°, 34 axial slices/volume, 150 volumes for the functional localizer, 138 volumes/run for the main experiment.

To determine individualized ROIs, an independent functional localizer task was adapted from Nath and Beauchamp (2012) study, which included five blocks of stimuli consisting of words presented visually and auditorily (five auditory-only and five visual-only in random order) of duration 20 s with 10 s of fixation baseline between each block. Each block contained 10 2-s trials with one word per trial. The participants were instructed to watch and listen to the stimuli carefully.

### fMRI Data Analysis

Analyses were performed using the Analysis of Functional NeuroImages software (AFNI; Cox, 1996). The data were analyzed individually following the procedures described below. Pre- to post-test changes were examined through two levels of analyses: ROI analysis and functional connectivity analysis.

**Figure 2:**
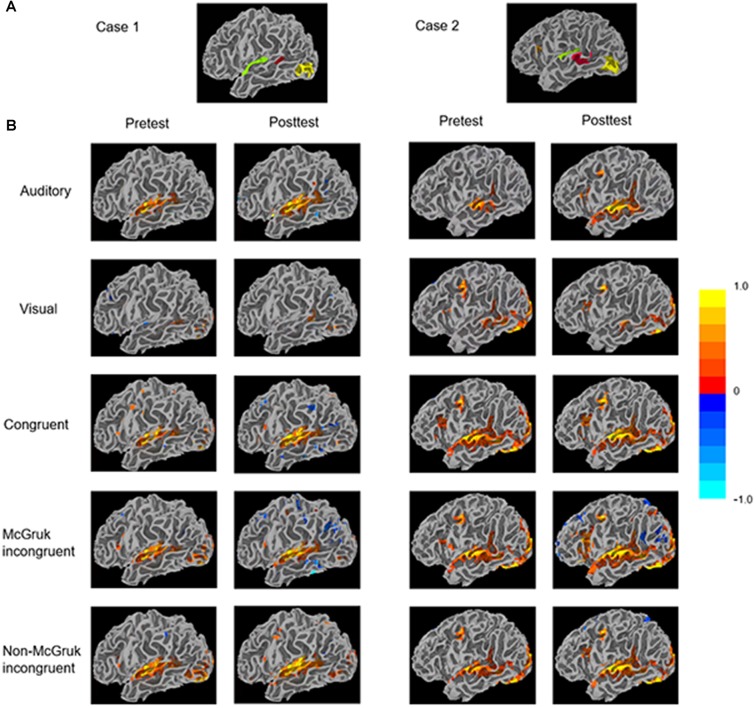
**(A)** Functionally-defined regions of interest (ROIs) identified through the functional localizer of the two cases. The audiovisual (AV) ROI (red) contains voxels responsive to both auditory and visual words in the posterior STS (pSTS). The auditory ROI (green) contains voxels responsive to auditory words within Heschl’s gyrus. The visual ROI (yellow) contains voxels responsive to visual words within extrastriate lateral occipitotemporal cortex. **(B)** The patients’ surface mapping showing activity in each condition. Clusters were identified through voxel-wise statistics corrected for multiple comparison using the False Discovery Rate algorithm with *q* (adjusted *p*) < 0.05.

All EPI data underwent standard preprocessing steps including registration to the T1-weighted anatomical scan, smoothing with a Gaussian blur of 4 mm FWHM, and distortion correction using FSL’s topup tool (Smith et al., 2004). Functional localizers from two sessions were combined for ROI definition (Figure 2). Specifically, clusters of significant voxels (corrected for multiple comparison using False Discovery Rate thresholding at *q* < 0.05) were used to functionally define ROIs for each participant separately within left hemisphere using FreeSurfer (Dale et al., 1999) and AFNI’s Surface Mapper (SUMA; Saad and Reynolds, 2012). Three ROIs were chosen based on previous literature on McGurk perception: the AV ROI included voxels responsive to both auditory and visual words in the posterior STS; the auditory (A) ROI included voxels responsive to auditory words only within Heschl’s gyrus; and the visual (V) ROI included voxels responsive to visual words only within extrastriate lateral occipitotemporal cortex.

Beta coefficients were first obtained using the General Linear Modeling (GLM) for each stimulus condition, scaled such that units were percentage signal change relative to the voxel mean, were averaged across voxels within each ROI. These mean beta values served as the dependent variables in ROI analyses. Then we performed voxel-wise functional connectivity analyses between the multi-sensory ROI and uni-sensory ROIs using a beta series method (Rissman et al., 2004) where the multi-sensory (AV) ROI served a seed time series.

To better quantify changes from pretest to posttest, individual level statistics were obtained by bootstrapping beta series within each ROI across trials for each condition. For example, to test if the auditory ROI in the AO condition showed significant change from pretest to posttest, we would resample the beta coefficients across trials for 1000× with replacement for pretest and posttest separately, and then compare if the distributions of the two test sessions differ significantly. Based on the overall pattern of increased activity and functional connectivity from pretest to posttest in both cases, one-tailed test with a significance level of 0.05 was used for the current case report (see Tables 2, 3).

**Table 2:** **Case 1 (hearing aid (HA) use) data showing activities of the three regions of interest (ROIs)—auditory ROI, visual ROI, audiovisual (AV) ROI, and functional connectivity between uni-sensory ROIs and AV ROI, in the five stimulus conditions at pretest and posttest**.

Condition	Auditory ROI			Visual ROI			AV ROI			Auditory-AV			Visual ROI-AV		
	Pre	Post	*p*	Pre	Post	*p*	Pre	Post	*p*	Pre	Post	*p*	Pre	Post	*p*
Auditory	0.39	0.43	0.528	−0.05	−0.15	0.704	0.23	0.39	<0.05*	0.19	0.72	<0.001***	0.18	0.47	<0.01**
Visual	−0.09	0.00	0.295	0.21	0.15	0.713	0.24	0.36	0.185	0.49	0.58	0.075	0.25	0.30	0.242
Congruent	0.38	0.49	0.260	0.19	0.11	0.293	0.33	0.38	0.189	0.49	0.58	0.221	0.24	0.39	<0.05*
McGurk incongruent	0.42	0.45	0.626	0.30	0.13	0.910	0.29	0.45	0.175	0.48	0.56	0.346	0.14	0.30	0.075
Non-McGurk incongruent	0.42	0.61	0.136	0.31	0.20	0.512	0.39	0.46	0.198	0.59	0.78	<0.01**	0.22	0.46	<0.01**

For each ROI, numbers in the first two columns present percentage signal change in activity relative to baseline at pretest and posttest; the third column presents significance of change in activity from pretest to posttest obtained from bootstrapping. For each pair of ROIs, numbers in the first two columns present connectivity measured by averaged correlation coefficient between voxels within the uni-sensory ROI and the AV ROI at pretest and posttest; the third column contains significance (p value) of change in functional connectivity from pretest to posttest obtained from bootstrapping. *p < 0.05; **p < 0.01; ***p < 0.001.

**Table 3:** **Case 2 (HA use + AV training) data showing activities of the three ROIs—auditory ROI, visual ROI, AV ROI and functional connectivity between uni-sensory ROIs and AV ROI, in the five stimulus conditions at pretest and posttest**.

Condition	Auditory ROI			Visual ROI			AV ROI			Auditory-AV			Visual ROI-AV		
	Pre	Post	*p*	Pre	Post	*p*	Pre	Post	*p*	Pre	Post	*p*	Pre	Post	*p*
Auditory	0.19	0.42	<0.05*	−0.06	−0.06	0.496	0.20	0.35	<0.01**	0.29	0.45	0.222	0.14	0.31	0.115
Visual	0.00	−0.09	0.991	0.26	0.20	0.875	0.24	0.20	0.768	0.09	0.29	0.056	0.14	0.15	0.255
Congruent	0.30	0.49	<0.001***	0.25	0.22	0.883	0.38	0.39	0.351	0.23	0.31	0.363	0.17	0.18	0.347
McGurk	0.25	0.42	<0.001***	0.24	0.25	0.334	0.32	0.44	<0.001***	0.19	0.39	<0.01**	0.09	0.17	0.099
incongruent
Non-McGurk	0.21	0.39	0.068	0.18	0.19	0.451	0.26	0.40	<0.01**	0.22	0.42	<0.01**	0.11	0.26	<0.01**
incongruent

For each ROI, numbers in the first two columns present percentage signal change in activity relative to baseline at pretest and posttest; the third column presents significance of change in activity from pretest to posttest obtained from bootstrapping. For each pair of ROIs, numbers in the first two columns present connectivity measured by averaged correlation coefficient between voxels within the uni-sensory ROI and the AV ROI at pretest and posttest; the third column contains significance (p value) of change in functional connectivity from pretest to posttest obtained from bootstrapping. *p < 0.05; **p < 0.01; ***p < 0.001.

## Results

### Case 1

The only significant change of ROI activity from pretest to posttest was in the AO condition that activity within the AV ROI significantly increased from pretest to posttest (AV: *p* < 0.05; Table 2 and Figure 2).

In functional connectivity analysis, both uni-sensory ROIs became significantly more synchronized with the multi-sensory ROI from pretest to posttest in the AO condition (A-AV: *p* < 0.001; V-AV: *p* < 0.01). However, due to the fact that the visual ROI showed barely positive activation in the AO condition, the observed V-AV connectivity in this condition might just reflect an artifact of increased activity in the AV ROI instead of functional connectivity change between the two ROIs. Similarly, in the VO condition, the trend of increasing synchronization between the auditory ROI and the AV ROI from pretest to posttest (A-AV: *p* = 0.075*)* might just reflect slight stimulus-driven changes in the same direction in both ROIs. In the AV congruent condition, only the visual ROI became significantly more synchronized with the AV ROI from pretest to posttest (V-AV: *p* < 0.05). In the McGurk incongruent condition, only the visual ROI displayed a trend of increased synchronization with the AV ROI from pretest to posttest (V-AV: *p* = 0.075). In the non-McGurk incongruent condition, both uni-sensory ROIs became significantly more synchronized with the AV ROI (A-AV: *p* < 0.01; V-AV: *p* < 0.01).

### Case 2

In the AO condition, activities in the auditory ROI and the AV ROI showed significant increase from pretest to posttest (A: *p* < 0.05; AV: *p* < 0.01) with no significant change in the visual ROI (Table 3 and Figure 2). In the AV congruent condition, activity in the auditory ROI increased significantly from pretest to posttest (A: *p* < 0.001) with no significant change in the visual and AV ROIs. In the McGurk incongruent condition, activities in the auditory ROI and the AV ROI showed significant increase from pretest to posttest (A: *p* < 0.001; AV: *p* < 0.001) with no significant change in the visual ROI. In the non-McGurk incongruent condition, activity in the AV ROI increased significantly from pretest to posttest (AV: *p* < 0.01) and a trend of increased activity in the auditory ROI (A: *p* = 0.068) with no significant change in the visual ROI.

In the AO condition, no significant change in functional connectivity was observed. In the VO condition, the auditory ROI showed a trend of increased synchronization with the AV ROI (A-AV: *p* = 0.056). But again, this trend might simply reflect slight stimulus-driven changes in both ROIs going in the same direction rather than connectivity change. In the McGurk incongruent condition, the auditory ROI became significantly more synchronized with the AV ROI from pretest to posttest (A-AV: *p* < 0.01), and the visual ROI showed a trend of increasing synchronization with the AV ROI (V-AV: *p* = 0.099). In the non-McGurk incongruent condition, both uni-sensory ROIs became significantly more synchronized with the AV ROI (A-AV: *p* < 0.01; V-AV: *p* < 0.01).

## Discussion

### Case 1: Cortical Plasticity Associated with Hearing Aid Use

The results showed that C1’s AV ROI became more responsive during listening to AO syllables after HA use. This finding is novel as our report is the first to examine effects related to HA use from the perspective of AV speech perception or neural plasticity involving multi-sensory integration. Moreover, whether there is “acclimatization” effect in terms of change in electrophysiological responses to acoustic input after HA use still bares controversies (Dawes et al., 2014). We suggest that the observed enhancement in the STS following HA use might reflect an increased tendency in matching the speech sounds with corresponding abstract phonological representations in multi-sensory forms (Barraclough et al., 2005). Although speculative, this finding reminds us to consider the role of multi-sensory representation of speech sounds in aural rehabilitation via amplification device. For example, adding visual cues to speech signals benefits elderly HA users but not elderly normal hearing listeners during speech identification (Moradi et al., 2016).

Functional connectivity results showed more pervasive effects across conditions. Specifically, all three AV conditions showed significant or suggestive increase of V-AV connectivity after HA use. Study of AV perception has shown that the modality with higher S/N tended to show greater connectivity with STS compared to the modality with lower S/N (Nath and Beauchamp, 2011). Note that C1 had severe hearing loss over higher frequencies, which means input from the visual modality can be more reliable to him than input from the auditory modality. Given that HA users oftentimes rely on visual cues in noisy environment, the observed increase in V-AV connectivity might reflect a greater perceptual cue weighting of visual information for AV speech processing as an adaptive strategy to HA use.

Moreover, in the non-McGurk incongruent condition with visual /ba/ and auditory /ga/, the A-AV connectivity was also strengthened. In this condition, although the auditory and visual cues were unmatched, there was low fusibility between the two modalities because the auditory cue typically dominate the percept (listeners will hear auditory /ga/ despite the visual /ba/). Therefore, the strengthened A-AV connectivity may suggest more efficient use of auditory cues under auditory-dominant listening situation due to adaption to acoustic amplification through HA.

### Case 2: Cortical Plasticity Associated with Hearing Aid Use and Rehabilitative Training

This patient showed significant or suggestive increase in activity within the auditory ROI from pretest to posttest in all conditions except for the VO condition. This pattern may reflect greater involvement of the auditory modality in response to acoustic signals after HA use and auditory training. In addition, the AV ROI showed significantly increased responsiveness to the AO syllables, McGurk incongruent syllables and non-McGurk incongruent syllables, which might indicate a greater tendency of matching the speech sounds with corresponding abstract phonological representations in multi-sensory forms when visual cue is not available or when AV incongruity is present.

Functional connectivity results revealed a clear pattern that the uni-sensory ROIs became more synchronized with the multi-sensory ROI from pretest to posttest in the two AV incongruent conditions. Recall that in the RMQ training, the presence of noise forces the listener to rely on lip movement for successful speech understanding. The current observation of enhanced A/V—AV connectivity might indicate that uni-sensory modalities were involved to a greater extent with the AV integration mechanism in the presence of AV incongruity, which might be associated with the explicit practice of speechreading through the RMQ training in addition to adaptation to HA use.

In addition to the fMRI data, we have sought to examine behavioral plasticity through the Multimodal Lexical Sentence Test for Adults (MLST-A; Kirk et al., 2012), however, neither of the listeners showed noticeable improvement from pretest to posttest (see Table S1 in Supplementary Material), suggesting a potential dissociation between neural plasticity and behavioral plasticity measured by the MLST-A. For those interested, the current cases were part of a larger-scale behavioral study with similar finding on behavioral plasticity at a group level (Rishiq et al., 2016).

The current two-case report adds to the literature that has consistently demonstrated substantial brain plasticity induced by auditory training (including musical training) across the lifespan beyond the early sensitive period of learning (Zhang and Wang, 2007; Anderson and Kraus, 2013; Penhune and de Villers-Sidani, 2014; Yotsumoto et al., 2014). In particular, our fMRI data provided insights to the neural plasticity related to HA use and auditory training, as well as the role of AV integration in the rehabilitation process. The experimental design with identical protocols allowed us to examine the pre-to-post changes with each participant as their own baseline, which allowed fine-grained comparison at individual level. Despite the analytical approach and novelty of the findings, we need to acknowledge the limitations of the current case report. First, it is not able to tease apart the effects related to HA use and auditory training given the overlapping timeline of the two treatments. Second, speculative interpretation of results should be noted. For instance, potential residual hearing of the trained C2 might have contributed to her responsiveness to HA and auditory training. As the two volunteer subjects did not match in subject characteristics such as age, gender, degrees of hearing loss, it is impossible to make direct comparisons. Given the nature and scope of the current case report, we need to exercise caution and not overgeneralize the findings about cortical plasticity associated with HA use and AV training.

## Conclusion

This is the first fMRI report that has examined neural plasticity associated with HA use and auditory training targeting AV speech processing. Our data provide the initial evidence of cortical plastic change involving auditory cortex, STS and functional connectivity between auditory and visual regions and STS from two patients. As auditory training has been shown to be an effective rehabilitative tool that can potentially optimize speech processing and systematically improve speech communication in elderly individuals (Pichora-Fuller and Levitt, 2012; Ferguson and Henshaw, 2015; Morais et al., 2015), future investigation is warranted to investigate the neural basis for the short-term and long-term effects of specific auditory training protocols and the real world benefits. Our case report results underscore the malleable brain functionality of elderly hearing-impaired people, and AV speech perception as a topic for future research and practice in aging neuroscience and aural rehabilitation.

## Author Contributions

YZ and AR conceived this study. LY, AR, PCB and YZ designed the study. DR and HA recruited participants. LY, AR, PCB collected data. LY and PCB analyzed data. LY prepared the first draft and all co-authors contributed to writing the manuscript.

## Conflict of Interest Statement

The authors declare that the research was conducted in the absence of any commercial or financial relationships that could be construed as a potential conflict of interest.

## Footnotes

^1^http://sensesynergy.com/
