# Supplementary material for: Neuromodulatory Effects of Auditory Training and Hearing Aid Use on Audiovisual Speech Perception in Elderly Individuals
Source: Front Aging Neurosci. 2017 Feb 21;9:30. doi: 10.3389/fnagi.2017.00030 (PMC5318380; doi:10.3389/fnagi.2017.00030)
Supplement: Supplementary file 1 [file Table_1.docx]

**Supplementary Material**

This document provides supplementary data (Table S1) for the case report paper titled “*Neuromodulatory Effects of Auditory Training and Hearing Aid Use on Audiovisual Speech Perception in Elderly Individuals*” submitted to *Frontiers in Aging Neuroscience* (authored by Yu, Rao, Zhang, Burton, Rishiq, & Abrams. Corresponding author: Yang Zhang, [zhanglab@umn.edu](mailto:zhanglab@umn.edu)).

**Table S1.** Speech comprehension in noise measured by the Multimodal Lexical Sentence Test for Adults before and after HA use (and RMQ training).

Auditory only Audiovisual

| Case | SNR | -5 dB | 0 dB | 5 dB | -5 dB | 0 dB | 5 dB |
| --- | --- | --- | --- | --- | --- | --- | --- |
|  | pre | 0.18 | 0.49 | 0.82 | 0.4 | 0.61 | 0.86 |
| C1: HA | post | 0.11 | 0.69 | 0.86 | 0.43 | 0.65 | 0.96 |
| C2: HA and RMQ | pre | 0.07 | 0.4 | 0.83 | 0.375 | 0.78 | 0.875 |
|  | post | 0.14 | 0.6 | 0.86 | 0.22 | 0.85 | 0.89 |

HA: hearing aid; RMQ: ReadMyQuips training

**References**

Yu, L., Rao, A., Zhang, Y., Burton, P.C., Rishiq, D., & Abrams, H. (2017). Neuromodulatory effects of auditory training and hearing aid use on audiovisual speech perception in elderly individuals. Frontiers in Aging Neuroscience, 9, 30.

Online access: <http://journal.frontiersin.org/article/10.3389/fnagi.2017.00030/>
